# Compulsory Latin for Medical Students

**Published:** 1916-12-16

**Authors:** 


### COMPULSORY LATIN FOR MEDICAL STUDENTS.

It is not often that a discussion in the General Medical Council has an attractive interest outside the bounds of the medical profession. The public welfare is indeed often concerned in these discussions, but the relation between the two is too remote to excite attention. The Council has to be content to do good by stealth and runs no risk of the blushes provoked by fame. The recent debate on the question whether Latin should or should not be a compulsory subject in the medical preliminary examination is in some measure an exception to this rule. Obviously the topic is one which is allied to large educational issues, and some of these have been pushed to the forefront by the European struggle and by the kindred question of national efficiency. Again, Latin as an essential part of medical education has a traditional appeal, and a proposal to uproot it excites strong feelings in the more conservative type of mind. These influences may be readily recognised in the recent discussion, and they lent to the discussion a degree of feeling which is somewhat unusual in the Council Chamber.

To some extent the Council has had the ground cut from under its feet. Five of the English universities, including the University of London, no longer require Latin as an essential element in their matriculation examinations. They declare, in other words, that a student may be fit to enter on university studies even though he is not equipped in this particular respect. Still more, a student who has entered a university in the manner just contemplated may proceed, at least in some instances, to graduation in Arts or Science without any examination achievement in Latin. Hence for the Medical Council to maintain the rule which has hitherto existed it would be necessary to hold that even Arts and Science graduates of certain of the universities could not be regarded as fit to enter on the study of medicine. This proposition is a very large one, and it would be difficult to maintain, especially in view of the more liberal educational methods of the day and hour. If the responsibility of the Council in this respect is to secure that the student has received a sufficient mental training to enable him to engage successfully in medical study, it surely must be satisfied with his possession of a university degree, or even with a certificate of matriculation. That Latin may be an instrument by which this training can be effected is admitted, but the point at issue is whether it is an essential instrument, or whether, on the other hand, other subjects may not be entitled to equal recognition. Great stress is laid by those who love the ancient ways on the utilitarian value of Latin to the medical student and practitioner, and in this there is certainly some force. But the average equipment in this direction is a slender one, and although its disappearance might mean some loss, the position would not be without compensation if a higher standard of training in other directions were enforced. In our view this is the central point of the controversy. Importance, in so far as mental training is concerned, rests less with the subject of study than with the manner and spirit of the teaching. Here, and in the standard of thoroughness adopted, lie the influences which fit the mind for large views and severe tasks, and while no doubt omniscience would be a very desirable attainment, no modern student can profess to take more than a limited field for his province. Whatever be his future movement, it is in his early days that the student must be disciplined in methods of mental effort. That subjects other than Latin can, if rightly taught, effect this discipline we have no doubt, and our sympathies therefore are with the majority of the Medical Council.

